# Pricing and Reimbursement of Biosimilars in Central and Eastern European Countries

**DOI:** 10.3389/fphar.2017.00288

**Published:** 2017-06-08

**Authors:** Paweł Kawalec, Ewa Stawowczyk, Tomas Tesar, Jana Skoupa, Adina Turcu-Stiolica, Maria Dimitrova, Guenka I. Petrova, Zinta Rugaja, Agnes Männik, Andras Harsanyi, Pero Draganic

**Affiliations:** ^1^Institute of Public Health, Faculty of Health Sciences, Jagiellonian University Medical CollegeKraków, Poland; ^2^Department of Organisation and Management in Pharmacy, Faculty of Pharmacy, Comenius University in BratislavaBratislava, Slovakia; ^3^CZECHTA Institute o.p.s.Prague, Czechia; ^4^Faculty of Pharmacy, University of Medicine and Pharmacy of CraiovaCraiova, Romania; ^5^Department of Organization and Economy of Pharmacy, Faculty of Pharmacy, Medical UniversitySofia, Sofia, Bulgaria; ^6^Senior Expert at The National Health ServiceRiga, Latvia; ^7^Institute of Family Medicine and Public Health, University of TartuTartu, Estonia; ^8^National Health Insurance Fund of HungaryBudapest, Hungary; ^9^Department of Health Policy and Health Economics, Eötvös Loránd UniversityBudapest, Hungary; ^10^Croatian Agency for Medicinal Products and Medical DevicesZagreb, Croatia

**Keywords:** biosimilar pharmaceuticals, pricing, reimbursement, original products, interchangeability

## Abstract

**Objectives:** The aim of this study was to review the requirements for the reimbursement of biosimilars and to compare the reimbursement status, market share, and reimbursement costs of biosimilars in selected Central and Eastern European (CEE) countries.

**Methods:** A questionnaire-based survey was conducted between November 2016 and January 2017 among experts from the following CEE countries: Bulgaria, Czech Republic, Croatia, Estonia, Hungary, Latvia, Lithuania, Poland, Slovakia, and Romania. The requirements for the pricing and reimbursement of biosimilars were reviewed for each country. Data on the extent of reimbursement of biologic drugs (separately for original products and biosimilars) in the years 2014 and 2015 were also collected for each country, along with data on the total pharmaceutical and total public health care budgets.

**Results:** Our survey revealed that no specific criteria were applied for the pricing and reimbursement of biosimilars in the selected CEE countries; the price of biosimilars was usually reduced compared with original drugs and specific price discounts were common. Substitution and interchangeability were generally allowed, although in most countries they were at the discretion of the physician after a clinical assessment. Original biologic drugs and the corresponding biosimilars were usually in the same homogeneous group, and internal reference pricing was usually employed. The reimbursement rate of biosimilars in the majority of the countries was the same and amounted to 100%. Generally, the higher shares of expenditures were shown for the reimbursement of original drugs than for biosimilars, except for filgrastim, somatropin, and epoetin (alfa and zeta). The shares of expenditures on the reimbursement of biosimilar products ranged from 8.0% in Estonia in 2014 to 32.4% in Lithuania in 2015, and generally increased in 2015. The share of expenditures on reimbursement of biosimilars in the total pharmaceutical budget differed between the countries, with the highest observed value for Slovakia and Hungary and the lowest—for Croatia.

**Conclusions:** The requirements for the pricing and reimbursement of biosimilar products as well as the access of patients to biologic treatment do not differ significantly between the considered CEE countries. Biosimilar drugs significantly influence the reimbursement systems of these countries, and the expenditure on the reimbursement of biosimilars is increasing as they are becoming more accessible to patients.

## Introduction

Biologics are complex, protein-based drugs used in various diseases, including diabetes (insulins), inflammatory diseases, and cancers. They are produced in living organisms, such as bacteria, yeast, animals, or plants. The introduction of biologics has been a major breakthrough in the treatment of numerous conditions, but the drugs are often expensive (Mulcahy et al., 2014; Ornes, 2015). A biosimilar is a biological product that is approved for use on the basis of high similarity to an original biological product, known as a reference product or originator drug, and that shows no clinically meaningful differences in terms of safety and effectiveness from the original product. Only minor differences in clinically inactive components are acceptable, and a biosimilar provides the same clinical benefits as an original drug that is already on the market. Pharmaceutical companies can release biosimilar products after a patent on a particular biologic drug has expired. Over the past decade, over 80 biologic molecules have been launched globally (IMS, 2016). By 2020 the global market of biologic medicinal products is projected to exceed 390 billion USD and its value is estimated to account for up to 28% of the global pharmaceutical market. By this time, biosimilar products will have the potential to enter markets as follow-ons to a number of key biologics which have the current sales of more than 40 billion EUR. Almost 50 distinct biosimilars are currently in development (IMS, 2016). Biosimilars have the potential to reduce the cost of treatment similarly to generics in the case of traditional, chemically synthesized drugs. By ensuring greater access to the same substance at a lower cost, they will greatly affect the current situation of pharmaceutical and insurance companies, as well as patients themselves (Ornes, 2015; IMS, 2016). It was estimated that the cost-saving potential of biosimilars could exceed 44.2 billion USD over 10 years (Mulcahy et al., 2014). According to another source, the cost-saving potential of biosimilars in the European Union (EU) and the United States (US) could equal even more than 50 billion EUR over 5 years and reach even 100 billion EUR in 2020 (IMS, 2016).

Biologics are becoming increasingly important on the pharmaceutical market. They are listed among 10 key therapies in the world, and their sales volumes largely increased in the period from 2009–2014. In 2009 only 2 biologics were among 10 key therapies in the world: etanercept and infliximab (listed the sixth and seventh, respectively). But in 2014 there were already 5 biologics: adalimumab (first), insulinum glargine (second), etanercept (fifth), infliximab (seventh), and rituximab (tenth) (Long, 2015). In Germany the position of biologics is even higher: in 2014 the top 5 key therapies were biologics (adalimumab, bevacizumab, etanercept, trastuzumab, and rituximab), and there were 2 additional biologics, infliximab and interferon beta-1a, listed as the ninth and tenth, respectively. There were only 3 small-molecule products listed in the top 10. Such situation has been observed since 2009 when 5 out of the top 10 products were biologics (adalimumab, etanercept, interferon beta-1a, enoxaparin, and trastuzumab) (Long, 2015). Several biologics will lose patent exclusivity between the years 2015 and 2020 (see Table 1).

**Table 1:** **Patent expiry dates of biologics (Derbyshire, 2015)**.

**Biologics**	**Patent expiry date in the EU**	**Patent expiry date in the US**
Humira^®^ (adalimumab)	2018	2016
Avastin^®^ (bevacizumab)	2022	2019
Enbrel^®^ (etanercept)	2015	2028
Remicade^®^ (infliximab)	2015	2018
NovoLog^®^/NovoRapid^®^ (insulin aspart)	2011	2014
Lantus^®^ (insulin glargine)	2014	2014
Avonex^®^ (interferon beta-1a)	2015	2015
Tysabri^®^ (natalizumab)	2015	2015
Xolair^®^ (omalizumab)	2017	2017
Neulasta^®^ (pegfilgrastim)	2017	2015
MabThera^®^ (rituximab)	2013	2016
Lucentis^®^ (ranibizumab)	2022	2020
Herceptin^®^ (trastuzumab)	2014	2019

Sales of the 8 top-selling biologics which will lose patent protection by 2020 are enormous. They equalled 42.3 billion EUR in September 2015 in 5 EU countries (France, Germany, Italy, Spain, United Kingdom) and in the US (IMS, 2016) and 60.3 billion USD globally in the whole US and EU (Long, 2015).

The introduction of biosimilars has increased the access to effective treatment by reducing costs, thus significantly increasing the use of biologics. The increase was the highest in countries with a previously limited access to these drugs, such as Romania, Bulgaria, and Czech Republic (IMS, 2016). The average price reduction of biologics due to introduction of biosimilars was 27% in the EU in general, and 50% in Romania, Bulgaria, and Czech Republic. The average increase in sales was 16 and 263%, respectively (IMS, 2016). The introduction of biosimilars has also increased the number of available treatment options.

In Europe, there is no universal approach to the reimbursement of biosimilars. Their pricing and the extent of price reduction can vary significantly between countries. The highest price reduction of biosimilars compared to original drugs was observed in Germany (55%) and France (39%), while it was lower in Scandinavian countries, ranging from 25 to 29% (IMS, 2016).

The aim of our study was to review the requirements for the reimbursement of biosimilars in selected CEE countries as well as to perform relative comparisons to assess differences in the market share and reimbursement costs of biosimilars between those countries.

## Materials and methods

The study was conducted in a period from November 2016 to January 2017. Experts from participating countries including Bulgaria, Czech Republic, Croatia, Estonia, Hungary, Latvia, Lithuania, Poland, Slovakia, and Romania provided in-depth data, valid for sophisticated assessment of biosimilars environment in reference countries, their pricing and reimbursement policy of biologics in their countries, as well as data on coverage for specific biologics, separately for original drugs and biosimilars. In order to facilitate comparisons between countries, reviews were based on a standardized questionnaire which included all necessary aspects covered by the project.

In our study, at least one leading national expert (scientist or government official) participated, who was involved in biosimilars pricing and reimbursement policy, representing each of considered countries; collection as well as assessment of data was conducted under supervision of coordinator of the whole project.

The first part of the survey included questions on the pricing and reimbursement of biologics in a given country, while the second part required providing data on the reimbursement status and level for each individual biologic drug. Reimbursement status data are valid for the fourth quarter of the year 2016; data on reimbursement costs of biologic drugs (separately for original drugs and biosimilars if country-specific data was available) in the years 2014 and 2015 were also collected along with the corresponding data on the total pharmaceutical and public health care budgets.

In our analysis, we included all currently approved biosimilars (25 product names) for 10 biologic drugs: enoxaparin sodium, epoetin alfa, epoetin zeta, etanercept, filgrastim, follitropin alfa, infliximab, insulin glargine, somatropine, and teriparatide according to the European Medicines Agency (EMA) official website (European Medicines Agency, 2017).

The questionnaires were distributed and responses were collected by e-mail. E-mail was also used to contact respondents in case of any doubts or additional questions.

The statistical analysis of obtained data was performed. The following values were calculated: the share of expenditure on the reimbursement of biosimilars and original drugs separately for each biologic and for each country, the share of expenditure on reimbursement of biosimilars as percentage of the total pharmaceutical budget and total health care budget in the year 2014 and 2015.

## Results

### A review of pricing and reimbursement of biosimilars in CEE countries

Selected issues relating to the reimbursement and pricing of biosimilars in the CEE countries were summarized in Table 2. None of the countries were shown to have a specific price negotiation procedure exclusively for biosimilars, except Lithuania, which applied a specific pricing pathway for outpatient care.

**Table 2:** **Pricing and reimbursement of biosimilars in selected Central Eastern European countries**.

**Question**	**Slovakia^a^ (Ministry of Health, 2011)**	**Estonia**	**Bulgaria**	**Latvia**	**Croatia**	**Lithuania**	**Romania**	**Poland (Kawalec and Malinowski, 2016; Panteli et al., 2016)**	**Czech Republic^b^**	**Hungary (ESzCsM Decree, 2017)**
Specific pricing pathway	No	No	No	No	No	Yes, for outpatients	No	No	No	No
Specific price discount	20%	15%	Yes, the value of discount is confidential	First–30%, second and third–10%, subsequent–5%	First–15%, subsequent–10%	First–30%, subsequent–15%	20%	25%	15% (30% during 2017 with change in legislation)	First–30%, second and third–10%, subsequent–lower ex-factory price as the already reimbursed cheapest product
Specific reimbursement criteria	No	No	No	No	No	No	No	No	No	No
Part of tenders	Yes	Yes, only in hospitals	Yes	No	No	Yes, only in hospitals	No	Yes	Yes	Yes
Substitution	Yes, at physician's discretion	Yes, at physician's discretion	Yes, at physician's discretion	n/a	Yes, at physician's discretion	n/a	Yes, at physician's discretion	n/a	Yes, at physician's discretion	Yes, obligatory in newly treated patients
Interchange ability	Yes, at physician's discretion	Yes, at physician's discretion	Yes, at physician's discretion	Yes	Yes, at physician's discretion	n/a	Yes, at physician's discretion	Yes, at physician's discretion	Yes, at physician's discretion	Yes, at physician's discretion
HTA evaluation	If original drug is reimbursed, there is no need for a full HTA documentation for biosimilars	Full HTA documentation is required	If original drug is reimbursed, there is only a Pharmaco-economic evaluation of biosimilars	Economic analysis and BIA	Only BIA is required, in some cases–full HTA	HTA is not required	Full HTA is required	If original drug is reimbursed, there is no need for a full HTA documentation for biosimilars, only simplified version of BIA	If original drug is reimbursed, there is no need for a full HTA documentation for biosimilars	Full HTA is required or simplified procedure, depends on the ex-factory price level
Timelines for reimbursement decision	180 days	First drug: 180 days; subsequent drug: 90 days	First drug: 90 days for HTA Committee recommendation and 90 days for reimbursement decision from the National Council on Prices and reimbursement, next drug: 60 days	180 days	90 days	n/a	90 days	180 days	165 by law, 200 days in reality	Normal procedure: 90 days; simplified procedure: 60 days
Homogeneous group	Yes	Yes	n/a	n/a	Yes	Yes	Yes	Yes	Yes	Yes
Internal reference pricing	Yes	Yes	Yes	n/a	Yes	Yes	Yes	Yes	Yes	Yes

*HTA, Health Technology Assessment; BIA, Budget Impact Analysis; n/a, not available*.

a *[Act No. 363/2011 Coll. on the Scope and Conditions of Payments for Medicines, Medical Devices and Dietetic Foods from Public Health Insurance and Amending Certain Acts, as Amended] Zákon ĉ. 363/2011 Z. z. o Rozsahu a podmienkach úhrady liekov, zdravotníckych pomôcok a dietetických potravín na základe verejného zdravotného poistenia a o zmene a doplnení niektorých zákonov, (2011) [cited 2016 Nov 07]. Available online at: http://www.zakonypreludi.sk/zz/2011-363 (Accessed February 13, 2017)*.

b *Act 48/1997 Coll., Legal act on Public Health Insurance, Amending some Related Laws. Czech Republic*.

We found specific price discounts for the first or subsequent biosimilars (or both) submitted for reimbursement in all countries. The discount ranged from 5 to 30% of the price of the original drug. In Hungary, the first biosimilar entering the market has to offer a price reduction of 30% in relation to the ex-factory price of the original product (ESzCsM Decree, 2017), the second—an additional 10% reduction of the ex-factory price of the first biosimilar product, and the third—a further 10% reduction of the ex-factory price of the second biosimilar product. Any additional product has to enter the market with a lower ex-factory price than the cheapest reimbursed product.

The reimbursement criteria for biosimilars were generally similar to those for other generic products, and there were no specific criteria exclusively for biosimilars in any of the countries (see Table 2). In Bulgaria the criteria for reimbursement were as follows: availability of an alternative medicine for the treatment of the disease for which the medicinal product is indicated, efficacy and therapeutic effectiveness, safety, appropriate pharmacoeconomic indicators, as well as a positive reimbursement status in at least 5 other EU countries^1^,^2^. In Romania each evaluation criterion had its own number of points, and the criteria were as follows: health technology assessment (HTA) based on therapeutic benefit (max. 15 points), HTA based on number of EU countries with a positive reimbursement status (max. 25 points), Positive Assessment Report issued by the National Agency for Medicines and Medical Devices (max. 45 points), and therapy costs—direct costs (max. 30 points). The maximum was 145 points and conditional reimbursement is for 60–79 points (Ministry of Health, 2013, 2014).

The price of biosimilars is regulated by the tendering system/procedure in the majority of the countries, except for Estonia and Lithuania where it applies only to inpatient care. In Slovakia, Poland, and Hungary, the main criterion for tendering is price. In Czech Republic, tenders are rarely established by the State Institute for Drug Control (SUKL) or health insurance funds, because there is a mandatory reassessment of the reimbursement level for all products within a reference group, based on the tender offer. This means that following a tender offer all products within the reference group will undergo a reassessment (usually a reduction) of reimbursement based on the tender price. However, tenders are frequently used for inpatient drugs. In Latvia, Croatia, and Romania, tendering is not used for biosimilars.

Therapeutic substitution, which is a change of one substance (e.g., infliximab) for another substance (e.g., adalimumab) with a similar therapeutic effect and indication, was allowed only at the discretion of the physician, except for Lithuania, Latvia and Poland, where substitution was not allowed. Interchangeability, which means using one biosimilar in place of another one [e.g., Remsima (infliximab) in place of Inflectra (infliximab)], was generally allowed but only at the discretion of the physician. The exception was Lithuania where interchangeability was not allowed. No legal basis for therapeutic substitution or interchangeability of biosimilars was reported for any of the countries.

If a given biosimilar is the first drug with a particular active substance to be reimbursed, a full HTA dossier is obligatory in all countries except Lithuania. If the original drug or another biosimilar with the same active substance has already been reimbursed, usually there is no need for full HTA documentation; in Poland only simplified Budget Impact Analysis (BIA) should be submitted (Kawalec and Malinowski, 2016; Panteli et al., 2016). In Bulgaria, if there is already the same International Non-proprietary Name (INN) on the Positive Drug List, the biosimilar product should be evaluated by the National Council on Prices and Reimbursement, providing the final reimbursement decision. In Croatia only BIA is required for biosimilars, and in certain cases, the Croatian HTA agency performs HTA evaluation.

The timelines for pricing and reimbursement decisions differed between the countries and ranged from 60 to 200 days, but in the majority of the countries, a 180-day deadline for decision-making process was applied according to the EU Transparency Directive. In Hungary (ESzCsM Decree, 2017), it depends on the type of the procedure applied: in the case of a standard procedure, it usually takes 90 days to make a reimbursement decision, and in the case of a simplified procedure—only 60 days. In Bulgaria since 2016 the procedure for new INNs is extended to 180 days as in the reimbursement decision process a HTA Committee is involved. Within 90 days the HTA Committee should issue recommendation for inclusion in the Positive Drug List. If the decision is positive, the National Council on Prices and Reimbursement of Medicinal Products should issue the final reimbursement decision in 90 days. In case there is already the same INN on the Positive Drug List the National Council on Prices and Reimbursement should issue the reimbursement decision in 60 or 90 days like in Hungary and Estonia, respectively (Regulation of Ministry of Social Affairs, 2017).

In all countries, except Bulgaria and Latvia which did not provide specific information, biosimilars belong to a homogenous group of drugs. In Estonia, the homogenous group is made with a transitional period of 3 months. Internal reference pricing was used in all countries, except Latvia for which this particular data is not available.

### Reimbursement status of biologics

We analyzed the reimbursement status of selected biologic drugs for which biosimilars are available, separately for each country, and indication. The level of reimbursement (the percentage of the drug price covered by public payer) per drug and country is shown in Table 3.

**Table 3:** **Reimbursement status of biologics**.

**Active substance**	**Therapeutic area**	**Reimbursement rate [original/biosimilar] valid for fourth quarter of 2016**									
		**Slovakia^e^**	**Estonia^a^^,^^f^**	**Bulgaria^b^**	**Latvia**	**Croatia**	**Lithuania**	**Romania^c^ (Ministry of Health, 2015a,b)**	**Czech republic**	**Poland**	**Hungary^d^**
Filgrastim	Neutropenia Febrile neutropenia	−/100%	100%	−/100%	−/100%	−/100%	100/100%	−/100%	100/100%	lump sum or 100%/lump sum or 100%	100% (300HUF prescription fee per unit)/100% (300HUF prescription fee per unit)
Etanercept	Rheumatoid arthritis	100%/−	100%	75% or 100%/−	100%/−	100%/−	100%/−	100%/−	100%/100%	100/100%	100%/−
	Juvenile idiopathic arthritis	100%/−	100%	75 or 100%/−	100%/−	100%/−	100%/−	100%/−	100/100%	100/100%	100%/−
	Psoriatic arthritis	100%/−	100%	75 or 100%/−	100%/−	100%/−	100%/−	100%/−	100/100%	100/100%	100%/−
	Ankylosing spondylitis	100%/−	100%	75 or 100%/−	100%/−	100%/−	100%/−	100%/−	100/100%	100/100%	100%/−
	Axial spondyloarthritis	100%/−	100%	75 or 100%/−	−	100%/−	100%/−	100%/−	100/100%	100/100%	100%/−
	Plaque psoriasis	100%/−	100%	75 or 100%/−	−	100%/−	100%/−	100%/−	100/100%	100/100%	100%/−
Infliximab	Rheumatoid arthritis	75.9/96.6% −100%	100%	−/75 or 100%	100/100%	100/100%	100/100%	100/100%	100/100%	100/100%	100%
	Crohn disease	75.9/96.6% −100%	100%	−/75 or 100%	75/75%	100/100%	100/100%	100/100%	100/100%	100/100%	100%
	Ulcerative colitis	75.9/96.6% −100%	100%	−/75 or 100%	75/75%	100/100%	100/100%	100/100%	100/100%	100/100%	100%
	Ankylosing spondylitis	75.9/96.6% −100%	100%	−/75 or 100%	100/100%	100/100%	100/100%	100/100%	100/100%	100/100%	100%
	Psoriatic arthritis	75.9/96.6% −100%	100%	−/75 or 100%	100/100%	100/100%	100/100%	100/100%	100/100%	100/100%	100%
	Plaque psoriasis	75.9/96.6% −100%	100%	−/75 or 100%	−	100/100%	100/100%	100/100%	100/100%	100/100%	100%
Insulin glargine	Diabetes mellitus	81.7−93.7%/100%	100%/−	100/100%	100%/−	100/100%	100%/−	100/100%	100/100%	30/30%	50 or 100% (300 HUF prescription fee per unit)/−
Somatropin	Growth disturbance/growth hormone deficiency	67.7−100%/67.7−100%	100/100%	100/100%	100/100%	100/100%	100/100%	100/100%	100/100%	100/100%	100% (300HUF prescription fee per unit)/100% (300HUF prescription fee per unit)
Enoxaparin	Thromboembolic disorders of venous origin	77−100%/−	100%/−	100%/−	75%/−	100%/−	−/−	100%/−	100%/−	lump sum/lump sum	90%/−
Epoetin alfa	Anemia	−/100%	−/−	100/100%	−/−	−/100%	n/a	−/−	100/100%	−/100%	−/100% (300 HUF prescription fee per unit)
	Kidney failure	−/100%	−/−	100/100%	−/−	−/100%	n/a	100/100%	100/100%	−/100%	−
	Cancer	−/100%	−/−	100/100%	−/−	−/100%	n/a	−/−	100/100%	−/100%	−
Epoetin zeta	Anemia	−/−	−/−	−/100%	−/100%	−/100%	n/a	−/100%	100/100%	−/−	−/100% (300 HUF prescription fee per unit)
	Kidney failure	−/−	−/−	−/100%	−/100%	−/100%	n/a	−/100%	100/100%	−/−	−/−
	Cancer	−/−	−/−	−/100%	−/−	−/100%	n/a	−/100%	100/100%	−/−	−/−
	Blood transfusion	−/−	−/−	−/100%	−/−	−/100%	n/a	−/−	−/−	−/−	−/−
Follitropin alfa	Anovulation	56.6%−57.0/67.4−70.0%	50/50%	100/100%	100%/−	100/100%	n/a	−/−	100/100%	lump sum/lump sum	25 or 70%/25 or 70%
Teriparatide	Osteoporosis	97.28%/−	−/−	50 or 100%/−	50%/−	100%/−	n/a	100%/−	100%/−	−/−	90%/−

*HUF, Hungarian Forint; GIST, Gastrointestinal Tumor; “−,” not reimbursed; n/a, not available*.

a *There is no information on reimbursement status separately for biosimilars and original of filgrastim, etanercept, infliximab*.

b *In Bulgaria filgrastim and enoxaparin are reimbursed only for inpatient; epoetin afla and zeta are reimbursed for outpatient only for kidney failure, for other indications are reimbursed only for hospitals; follitropin alfa in Bulgaria is 100% reimbursed by the hospitals but one patient has 3 free attempts per 1 year; lower percent where available refers only for outpatient practice for all products; there is no difference in reimbursement of biologicals (originator and biosimilar)*.

c *In Romania filgrastim is reimbursed in HIV, cirrhosis, hepatitis B, C, and D, cancer, bone marrow transplant, liver/kidney transplant*.

d *There is no information on reimbursement status separately for biosimilars and original of infliximab*.

e *Available online at: http://www.health.gov.sk/Clanok?lieky201611; Slovakia*.

f *Available online at http://sm.ee/et/ravimite-hinnastamine-ja-huvitamine; Estonia*.

The biologic drugs were reimbursed in the majority of cases in all countries: filgrastim for neutropenia or febrile neutropenia; etanercept for rheumatoid arthritis, juvenile idiopathic arthritis, psoriatic arthritis, and ankylosing spondylitis; infliximab for all approved indications (except plaque psoriasis in Latvia); insulin glargine for diabetes mellitus; and somatropin for growth disturbance or growth hormone deficiency. Epoetin alfa and epoetin zeta was not reimbursed in Estonia, while in each other country epoetin alfa or zeta was financed from public funds in at least one indication (no data for Lithuania). Etanercept was not reimbursed in Latvia in the treatment of axial spondyloarthritis and plaque psoriasis. Enoxaparin for thromboembolic disorders of venous origin was reimbursed in all countries except Lithuania. Follatropin alfa and teriparatide are reimbursed in majority of countries—only in Romania follitropin alfa was not financed from public funds, and teriparatide was not reimbursed in Estonia and Poland (no data for Lithuania).

Inpatient drugs generally had 100% reimbursement (filgrastim, etanercept, infliximab, epoetin alfa, and zeta products), although for insulin glargine the reimbursement rates ranged from 30 to 100%. In Hungary filgrastim, somatropin, epoetin alfa, and zeta products were reimbursed in 100% but a prescription fee per unit of 300 HUF (about 1 EUR) was covered by the patient. Etanercept and infliximab products were reimbursed in 100% in 2014 and 2015 through itemized payment in inpatient care. The reimbursement of filgrastim was 100% in all other countries, except Poland, where it was diversified and depended on the way of reimbursement. In the case of ambulatory drugs, patients had to pay a lump sum for a package of the product, but reimbursement was 100% for inpatient drugs. The level of reimbursement for enoxaparin was 100% in most countries except Poland, where the patient paid a lump sum, Latvia and Hungary, where the patient had to pay 25 and 10% of the price, respectively. In Slovakia selected insulin glargine, somatropin, and enoxaparin products were fully reimbursed, and for the remaining products patients had to pay from 18.3 to 32.3% of the price. Follitropin alfa had different level of reimbursement across countries—from 25% in Hungary to 100% in Latvia, Croatia, Bulgaria, and Czech Republic. Similar situation was observed in case of teriparatide—the level of reimbursement varied from 50% (Bulgaria for outpatients, Latvia) to 100% (Croatia, Bulgaria for inpatients, Romania, Czech Republic).

### Reimbursement costs

**Figure 1:**
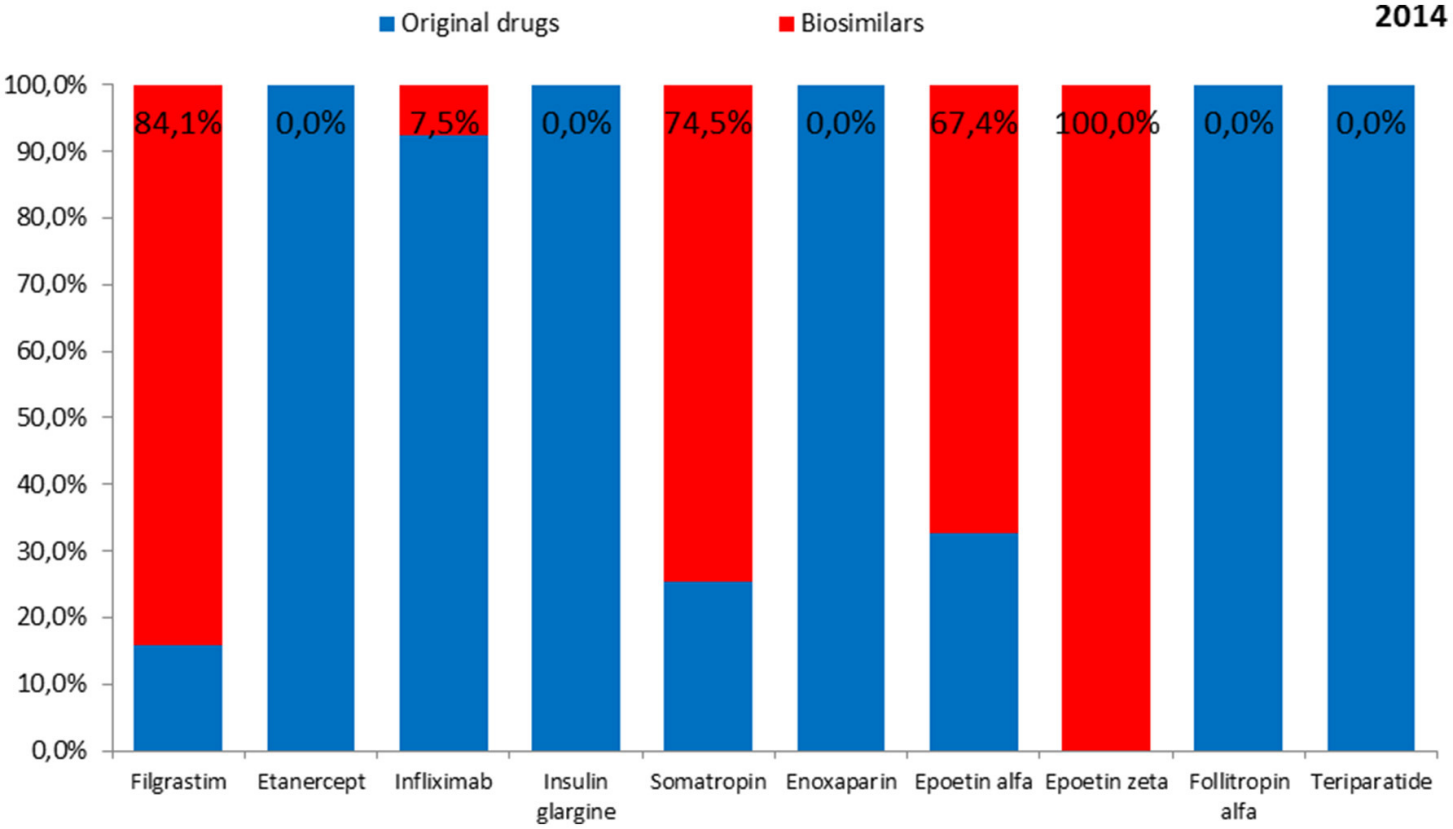
**The shares of expenditures for the reimbursement of biosimilars and original drugs for each biologic in 2014**.

**Figure 2:**
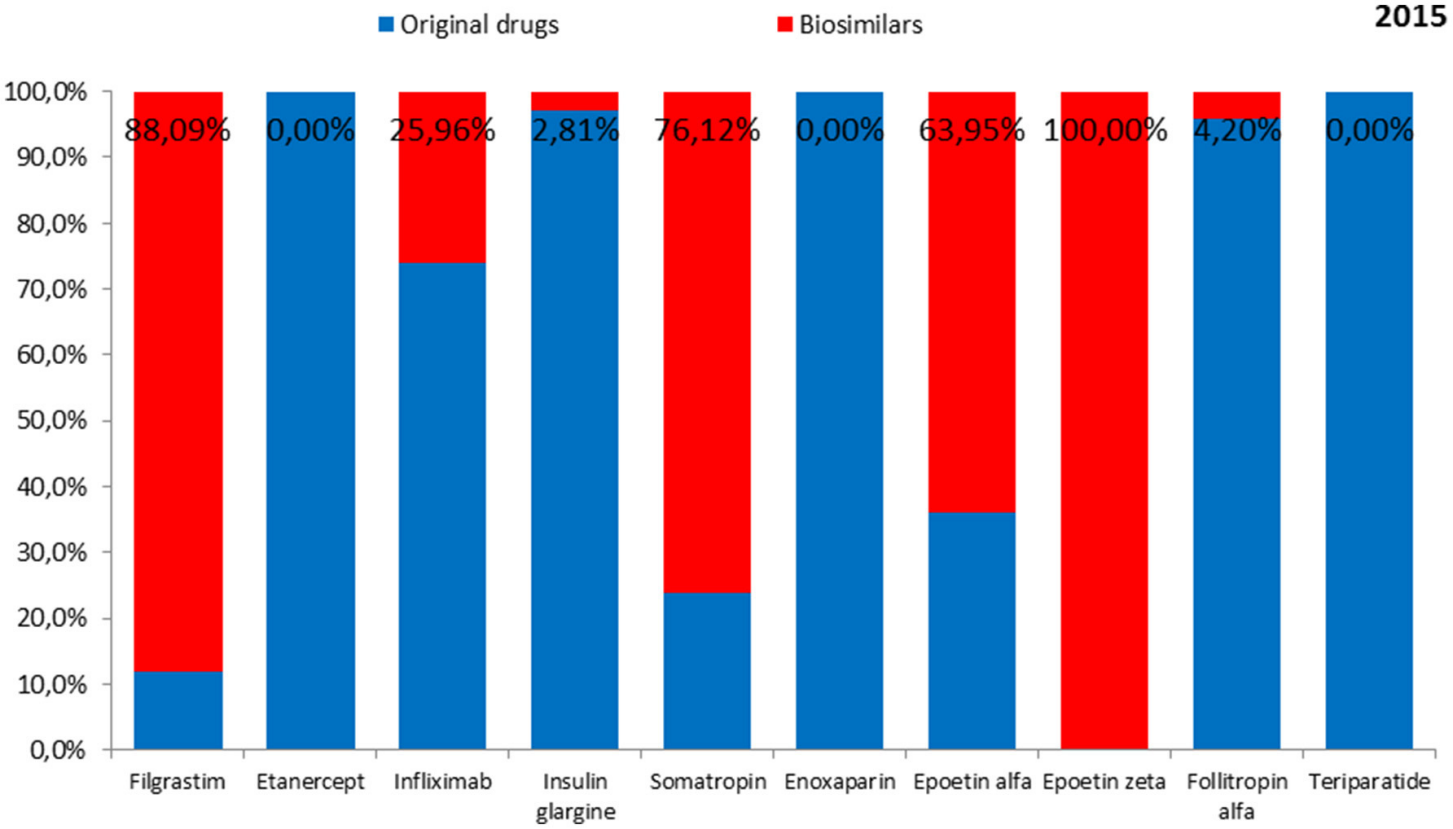
**The shares of expenditures for the reimbursement of biosimilars and originals for each biologic drug in 2015**.

The total expenditure on the reimbursement of biologic drugs in the CEE countries was 397,097,152 EUR in 2014 and 411,433,628 EUR in 2015. On average, 81.8% of the value in 2014 and 78.7% of the value in 2015 was covered by the reimbursement of original drugs. Romania and Bulgaria were excluded from this analysis owing to lack of information on expenditures according to the type of drug—original or biosimilar. The shares of expenditures on the reimbursement of biosimilars and original drugs for each biologic in the years 2014 and 2015 are shown in Figures 1, 2, respectively.

On average, the lowest expenditure was shown for the reimbursement of epoetin zeta—1.8 million EUR in 2014 and in 2015, and the highest—for enoxaparin (97.7 million EUR and 108.9 million EUR, respectively).

Biosimilars to etanercept, enoxaparin, and teriparatide were not reimbursed from public funds in any of the countries either in 2014 or 2015, while biosimilars to insulin glargine and follitropin alfa were not reimbursed in 2014. Therefore, no costs of reimbursement of those biosimilars were observed. Among original products, the lowest expenditure was shown for the reimbursement of epoetin alfa in the year 2014 (4.7 million EUR) and filgrastim in the year 2015 (4.0 million EUR) and the highest—for enoxaparin (97.7 million EUR in the year 2014 and 108.9 million EUR in the year 2015). Among biosimilars, the lowest reimbursement cost in 2014 was shown for epoetin zeta (1.8 million EUR) and in 2015—for follitropin alfa (0.5 million EUR). The highest reimbursement cost in 2014 and 2015 was shown for filgrastim (27.9 million EUR and 29.8 million EUR, respectively).

**Figure 3:**
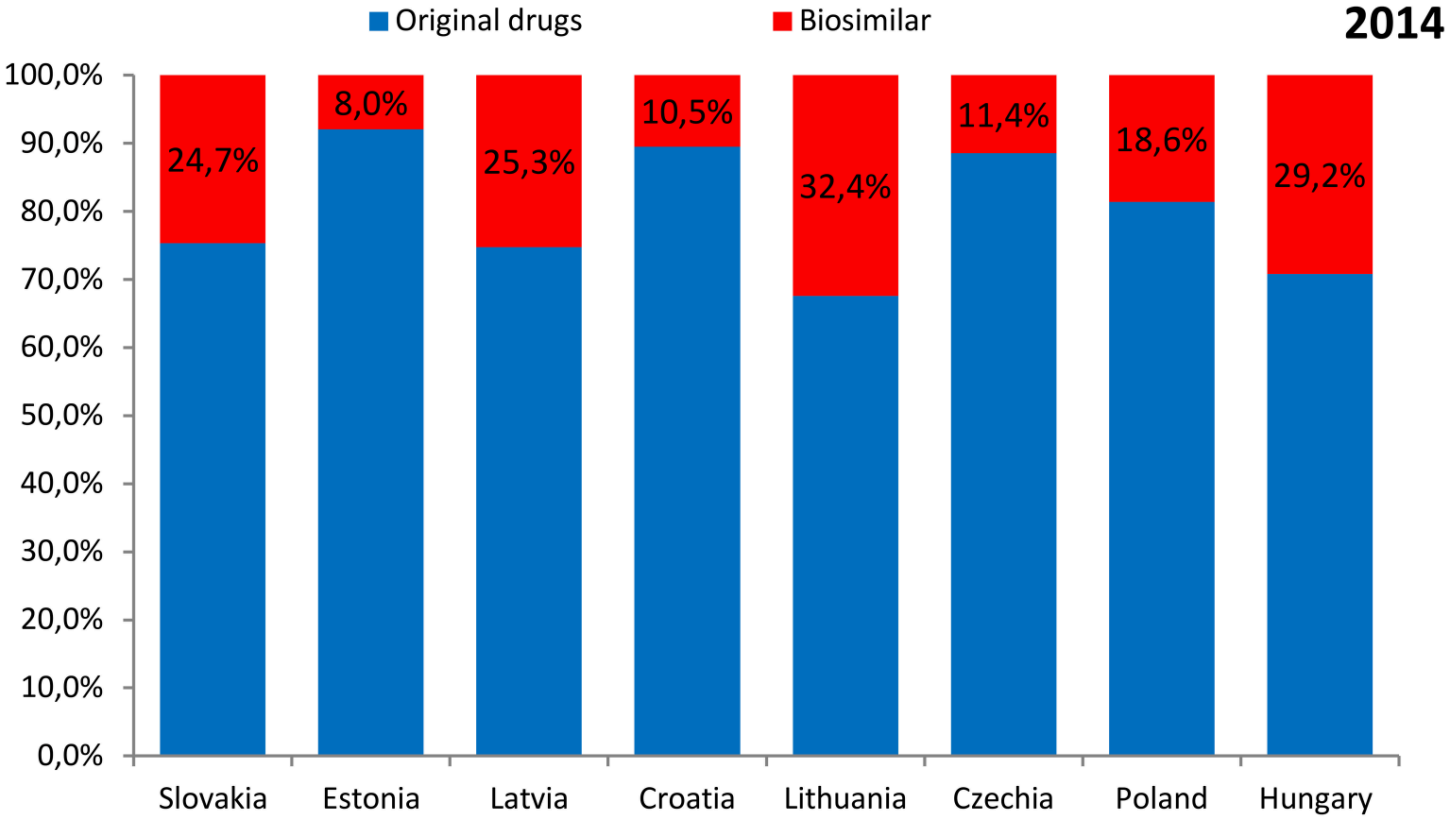
**The shares of expenditures on the reimbursement of biosimilars in individual countries in 2014**.

**Figure 4:**
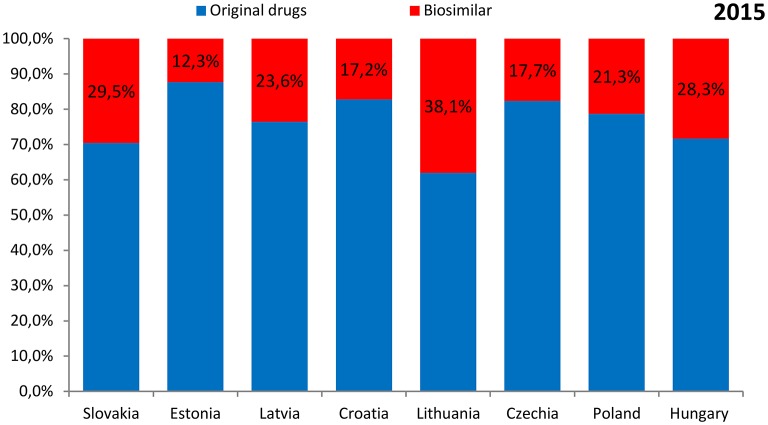
**The shares of expenditures on the reimbursement of biosimilars in individual countries in 2015**.

The shares of expenditures on the reimbursement of biosimilars in individual countries ranged from 8.0% in Estonia in 2014 to 38.1% in Lithuania in 2015. The data were presented separately for the years 2014 and 2015 in Figures 3, 4, respectively.

The highest expenditure on the reimbursement of biologic drugs was observed for Poland (117.0 million EUR in 2014 and 120.0 million EUR in 2015) and the lowest—for Romania (3.8 million EUR in 2014 and 3.7 million EUR in 2015). The highest expenditure on the reimbursement of original drugs was also observed for Poland (95.2 million EUR in 2014 and 94.5 million EUR in 2015), but the lowest—for Latvia (4.3 million EUR in 2014 and 5.1 million EUR in 2015). As for the reimbursement of biosimilars, the highest expenditure was revealed for Poland (21.8 million EUR in 2014 and 25.5 million EUR in 2015), and the lowest for Estonia (0.7 million EUR and 1.0 million EUR, respectively). Romania and Bulgaria were excluded from this analysis due to lack of information on expenditures according to the type of drug—original or biosimilar.

The total public pharmaceutical budget and total health care budget differed significantly between the countries. The lowest pharmaceutical and health care budgets were observed for Estonia and the highest—for Poland (see Table 4).

**Table 4:** **Total pharmaceutical and health care budgets in selected countries**.

**Country**	**Total public pharmaceutical budget [EUR]**		**Total public health care budget [EUR]**	
	**2014**	**2015**	**2014**	**2015**
Slovakia	884,000,000	905,000,000	3,882,000,000	3,995,000,000
Estonia	109,753,000	112,801,000	664,071,000	712,692,000
Bulgaria	416,168,185	436,107,447	2,037,894,823	2,030,737,139
Latvia	118,930,000	124,300,000	725,013,000	765,296,000
Croatia	667,500,000	702,300,000	3,190,000,000	3,080,000,000
Lithuania	245,000,000	253,000,000	1,380,000,000	1,470,000,000
Romania	1,838,000,000	1,774,000,000	4,892,000,000	4,775,000,000
Czech Republic	1,900,000,000	2,100,000,000	9,000,000,000	9,400,000,000
Poland	2,397,729,416	2,500,319,654	15,241,155,882	16,245,277,755
Hungary	935,359, 295	1,000,955,147	5,011,650,360	5,129,812,843

**Figure 5:**
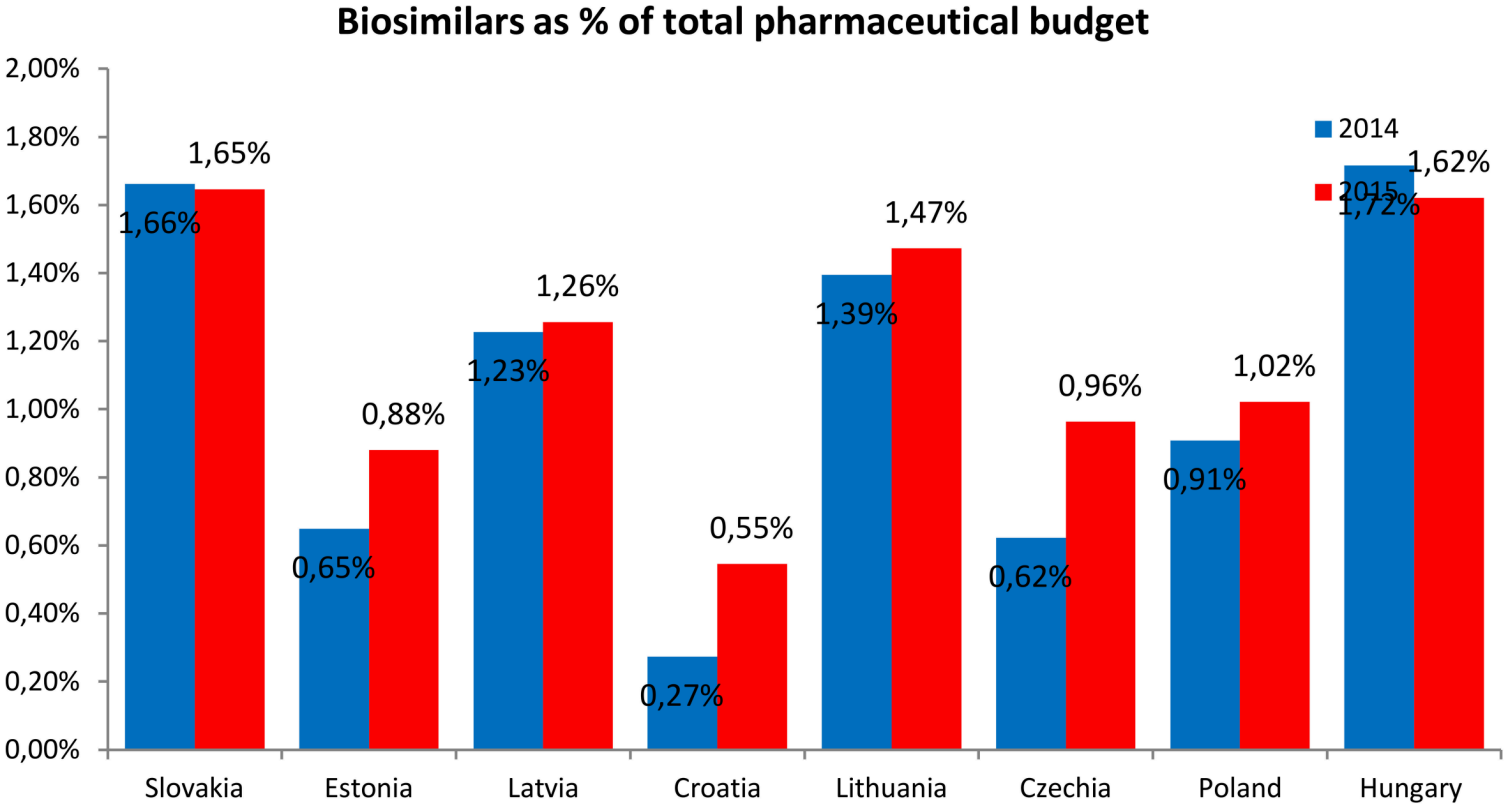
**Expenditures on the reimbursement of biosimilars as percentage of the total pharmaceutical budget**.

**Figure 6:**
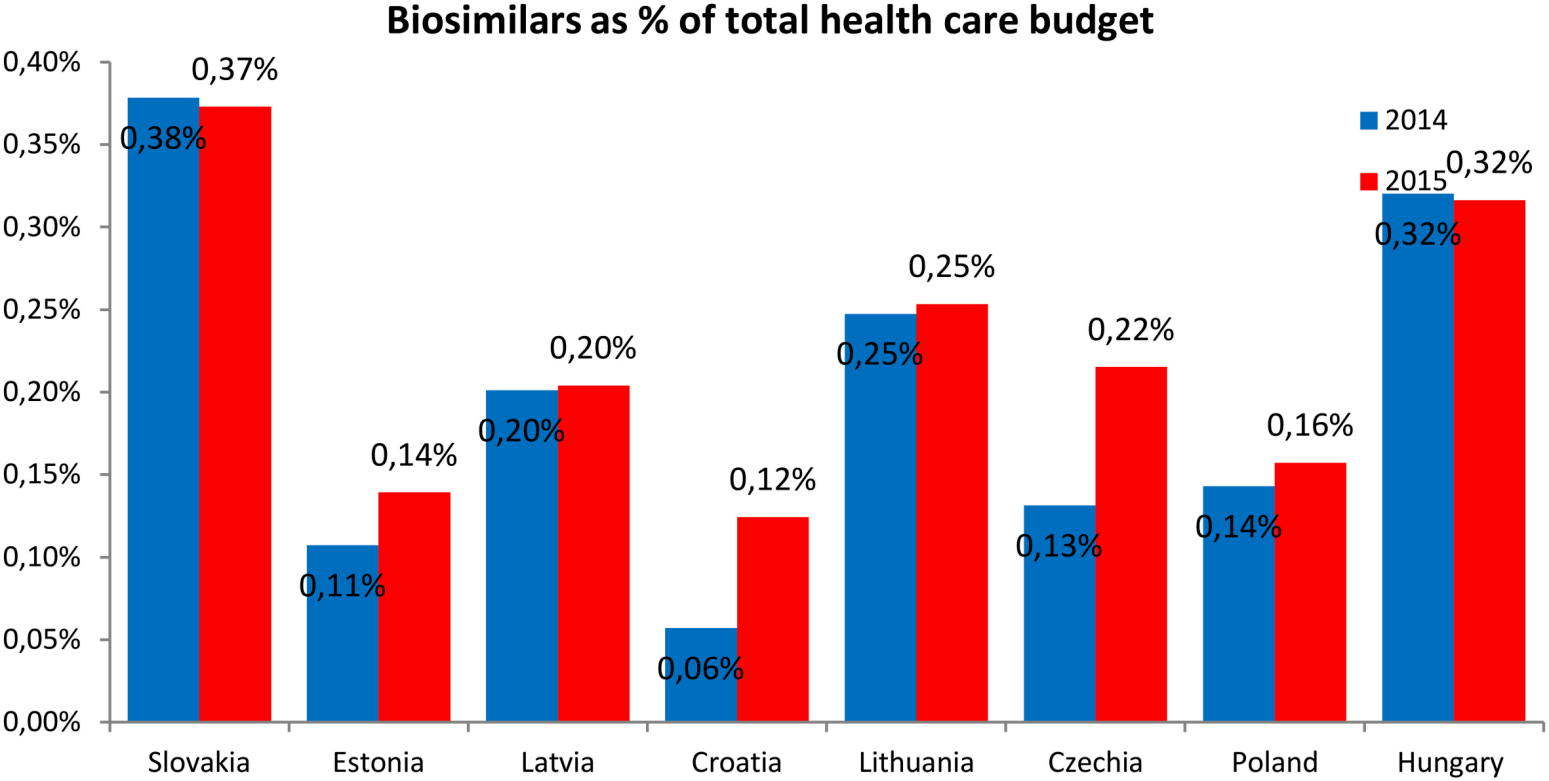
**Expenditures on the reimbursement of biosimilars as percentage of the total health care budget**.

The expenditures on the reimbursement of biosimilars were presented as the percentage of the total pharmaceutical budget (Figure 5) and percentage of the total health care budget (Figure 6).

The expenditures on the reimbursement of biosimilars constituted the highest share of the total pharmaceutical budget and total health care budget in Hungary and Slovakia (1.7 and 0.4%, respectively) and the lowest—in Croatia (0.3 and 0.1%, respectively). The expenditures on the reimbursement of biosimilars as the share of the total pharmaceutical budget were higher in 2015 than in 2014 in all countries, except Slovakia and Hungary where the shares were lower in 2015. The expenditures on the reimbursement of biosimilars as the share of the total health care budget were also higher in 2015 than in 2014 in all countries, again except Slovakia and Hungary where the expenditures were higher in 2014.

## Discussion

Biological drugs are highly effective but also very costly. The high price of original drugs limits access to treatment, especially in low-income CEE countries. The widespread use of biosimilars might significantly reduce the cost of biologic treatment, also for individual patients. In this study we identified biologic drugs that have their biosimilar counterparts. We also discussed issues relating to the reimbursement and pricing of biosimilars, as well as their reimbursement status in CEE countries.

Our review revealed that the access to biologic treatment within specific drug groups was similar but the level of the patient's co-payment for particular drugs differed between the CEE countries. For example in Bulgaria for 12 of the indications (out of 25) biological therapy is co-paid by outpatients thus becoming unaffordable in case of high prices. The use of biosimilars was quite common, and the percentage of biosimilars used in the treatment of specific disease groups increased during the study period. No reimbursement was observed for etanercept, insulin glargine (in 2014), follitropin alfa (in 2014), teriparatide, and enoxaparin, which may suggest that no biosimilars to those drugs were available in those countries. Only for filgrastim, somatropin, and epoetin (alfa and zeta) the reimbursement costs were higher for biosimilars than for original drugs. For the remaining drugs, the share of reimbursement costs of biosimilars in the total reimbursement costs of a biologic drug did not exceed 26%.

The shares of expenditures on the reimbursement of biosimilars in each country were similar and ranged from 8 to 32% in 2014 and from 12 to 38% in 2015. Expenditures on the reimbursement of biosimilars constituted from 0.3 to 1.7% of the total pharmaceutical budget in 2014 and was similar in the year 2015 (from 0.6 to 1.7%), but the average value was higher in the year 2015 (1.1% in the year 2014 compared with 1.2% in the year 2015). The shares of expenditures on the reimbursement of biosimilars in the total health care budget were also similar between the year 2014 and 2015—from 0.06% to 0.4% in 2014 and 2015, but again the average value was higher in the year 2015 (0.20% in the year 2014 compared with 0.22% in the year 2015).

In Hungary it is mandatory to use the cheapest available biological product in newly treated patients. For erythropoietin (EPO) and granulocyte colony-stimulating factors (GCSF), the so called biosimilar bids were introduced in 2012 (Hornyák et al., 2014). They are organized annually, and in each group of biologics the preferred drugs are those with the lowest price. As these products have 100% reimbursement, there is a prescription fee of only 300 Hungarian forints (HUF). Preferred drugs can be those with a price that is a maximum of 10% higher than the price of the reference product (i.e., the cheapest). Drugs with a price that is between 10 and 30% higher than that of the reference products are required to compensate the payer for the increased price with payback and are associated with a co-payment between 1,500 and 3,500 HUF. Increased co-payment is therefore a disincentive for patients to use these products. Drugs with a price that is 30% higher than that of the reference products are removed from the reimbursement list (i.e., positive list) 4 months after the biosimilar bid. Until then, they have a co-payment of 3,500 HUF. This allows some time for the patient and the treating physician to switch to a preferred product. Additionally, physicians are required to prescribe preferred biologics in a minimum 40% of the overall number of prescriptions.

In Estonia, inpatient drugs, such as filgrastim, infliximab, and etanercept, are reimbursed by the hospital, and it is hospital authorities who decide, usually through tendering procedure, which products and at what prices will be acquired. The data are usually confidential and hospitals do not have to officially report which products they use in provision of health care services. Therefore, there are limited data on the reimbursement costs for Estonia, both for original drugs and biosimilars.

In Romania, the rate of reimbursement for biosimilar products is 100% of the reference price, that is, in general, the lowest price of products with the same active substance, the same strength and the same pharmaceutical form (biosimilars) available for reimbursement. The physician can choose a specific product and the difference between the drug's price and the reference price is paid by patient, but in the case of biosimilars this difference is 0.

In Croatia the pricing and reimbursement process for biosimilars may sometimes require cost-effectiveness or cost-utility analysis^3^. In Latvia cost-minimization analysis and budget impact analysis are needed, while in Czech Republic an abbreviated clinical dossier should be presented, without economic evidence. In Czech Republic and Slovakia physicians prefer to treat new patients with biosimilars, while previously treated patients usually remain on the original product.

We performed a systematic review of medical databases (Medline via Pubmed and the Scientific Presentations database of International Society of Pharmaceoeconomics and Outcomes Research [ISPOR]) to identify relevant studies referring to biosimilar drug policy in CEE countries. However, we found no publications in line with the scope of our study, which emphasizes the novelty of our research. The review revealed only some papers on issues relating to the cost and reimbursement of biosimilars, but focusing mainly on the budget impact of biosimilar reimbursement or including a simplified discussion of an individual aspect (e.g., pricing) of biosimilar drug policy in individual CEE countries.

Hornyák et al. (2015) analyzed biosimilar bids of the Hungarian National Health Insurance Fund Administration (NHIFA) for colony-stimulating factor (CSF) products. Before the biosimilar bid, the NHIFA spent 7.49 billion HUF for health insurance reimbursement of CSF products, which decreased by 44% to 4.19 billion HUF in the first year after the bid. The analyses of the Hungarian price competition bid for biosimilar products showed a minor decline in the number of patients on treatment with CSF products, while the health insurance reimbursement of these drugs significantly decreased (Hornyák et al., 2014). Similar conclusions were drawn from the analysis of the biosimilar bids of the NHIFA for EPO products. The NHIFA spent 2.33 billion HUF for health insurance reimbursement of EPO products, which decreased by 47% to 1.23 billion HUF in the first year after the bid. Thus, the price competition bid of biosimilar products showed a health insurance reimbursement of these drugs to have significantly decreased (Nagy et al., 2015).

We also identified an abstract (Vogler et al., 2016) presenting the results of a survey analyzing the possible differences between pricing policies for generics and for biosimilar medicines in European countries including 25 EU countries (all except Ireland, Italy, and Luxembourg) as well as Albania, Belarus, Iceland, Norway, Serbia, Russia, Turkey, and Ukraine. While 23 of the 33 countries set the price of the generic in relation to the price of the originator, only 13 countries reported to do so for biosimilar medicines. Usually, the price difference between the biosimilar and originator medicine was set at a lower percentage rate than that between the generic and originator (e.g., 30%—generics, 15%—biosimilars in Croatia; 50%—generics, 30%—biosimilars in Lithuania; 35%—generics, 20%—biosimilars in Romania). It occurred that only Austria, Latvia, and Turkey applied the same price difference for generic and biosimilar medicines. The Netherlands have been tendering for generics in the outpatient sector during the last decade, but biosimilars have been included in tenders only recently. The authors concluded that European countries tend to apply similar pricing policies for generic and biosimilar medicines (Vogler et al., 2016).

Another study (Brodszky et al., 2014, 2016) analyzed the budget impact of introducing biosimilar infliximab for the treatment of patients with Crohn disease (CD) in the health care systems of 6 CEE countries: Bulgaria, Czech Republic, Hungary, Poland, Romania, and Slovakia. This budget impact model estimated the potential impact of biosimilar infliximab on health care budget over 3-year timeframe from the third-party payer perspective. The model tracked movement of the population of patients with CD between main states: (1) immune therapy, (2) infliximab, (3) biosimilar infliximab, and (4) adalimumab. Switching between biologics and biosimilar infliximab was also considered. A price difference of 25% was assumed for biosimilar infliximab compared to the originator. Budget impact was calculated as a difference in the total cost of scenarios with and without biosimilar infliximab. Over the 3-year period, with gradually interchanging 80% of infliximab to biosimilar, infliximab was expected to lead to a net benefit of 16,635,000 EUR compared with the scenario in which biosimilar infliximab would not be available. In the scenario in which interchangeability was not allowed, the budget savings amounted to 7,842,000 EUR. It was revealed that if budget savings were spent on the reimbursement of additional biosimilar infliximab treatments, ~722–1,530 additional patients with CD could be treated in the 6 countries (Brodszky et al., 2016). Based on these calculations, the introduction of biosimilar infliximab treatment for CD in CEE countries should bring about substantial cost savings or increase the number of patients with access to biologic therapy (Brodszky et al., 2014, 2016).

Rovira et al. (2013) described the development of biosimilars in 24 EU countries, Norway and Switzerland. Authors tried to identify the key parameters associated with biosimilars market dynamics and included three molecules as references: somatropin, epoetin, filgrastim. The results of this analysis showed that the market share of biosimilars for included molecules has risen rapidly in the years from 2007 to 2010. The key drivers responsible for market dynamics were: generics price control policy, countries' gross national income and expenditure on health, pharmacists' generics substitution and medicines' price level index. Introduction of biosimilars resulted in reduction of biologics' cost, however this reduction seems to be smaller than for conventional generics.

Authors of another study (Remuzat et al., 2017) also tried to identify the key drivers for market penetration of biosimilars in Europe. Ten EU member states were included in the analysis and 20 biosimilars. It was concluded, that incentive policies applied to biosimilars and biosimilars' uptakes were heterogeneous across analyzed countries. Incentive policies and the date of first biosimilar market entry were positively correlated with biosimilar uptake, while pharmaceutical expenditure per capita and the highest generic uptake were inversely correlated with biosimilar uptake. Also the average generic price discount over originator and the number of biosimilars seemed to influence the biosimilar uptake, however biosimilar price discounts had no impact on the uptake.

Grabowski et al. in their study (Grabowski et al., 2014) analyzed the experiences with biosimilars to epoetin alfa and filgrastim in 5 EU countries. One major finding was that although the EU has a common regulatory system for approving biosimilars, differences in reimbursement practices and incentives as well as variations in medical practices are observed across countries. It was observed that biosimilar price discounts were likely to be modest compared to generics. Another finding was that cost savings from the introduction of biosimilars in the European countries have been tempered by the fact that competition has been limited to the first-generation reference products. Dynamic competition through the market entry of next-generation biologics is an important consideration in analyzing the market impact of biosimilars and their potential savings to the health-care system.

In countries of the EU as well as outside the EU evaluations on costs of biologic drugs were also performed (Jakovljevic, 2014) but with no sophisticated analyses of market share and reimbursement costs between original biologics and biosimilars corresponding to results of our study.

## Conclusions

Our study revealed that biosimilars significantly influenced the reimbursement systems in the selected CEE countries. Expenditures on the reimbursement of biosimilars are increasing, and the access of patients to this type of treatment is improving. The share of expenditures on the reimbursement of biosimilars in the considered CEE countries increased during the study period, along with an increase in expenditures on the reimbursement of biosimilars as percentage of the total pharmaceutical budget. The considered CEE countries are similar in terms of the requirements for the pricing and reimbursement of biosimilars.

## Author contributions

PK conceived the conception and design of the study, including protocol and questionnaires preparation, and coordinated the project. PK contributed in acquisition of data. PK and ES carried out the data management, statistical analysis, interpretation of data and prepared the draft of the manuscript. TT, JS, AT, MD, GP, ZR, AM, AH, and PD collected and provided input data for corresponding countries. All authors contributed to editing the manuscript and approved the final version submitted for publication. PK is the guarantor.

### Conflict of interest statement

The authors declare that the research was conducted in the absence of any commercial or financial relationships that could be construed as a potential conflict of interest.

## Footnotes

^1^*Annex I and Annex II of the Positive Drug List of Bulgaria*. Available online at: www.ncpr.bg (Accessed February13, 2017).

^2^*Ordinance on the Terms, Rules and Procedure for Regulation and Registration of Prices for Medicinal Products/30.04.2013, Ammended and Supplemented, SG 8 of 24.01.2017*. Available online at: http://www.ncpr.bg/ (Accessed February13, 2017).

^3^Available online at: http://www.hzzo.hr/zdravstveni-sustav-rh/trazilica-za-lijekove-s-vazecih-lista/arhiva-liste-lijekova (Accessed February13, 2017).
